# Activated Protein C Has a Protective Effect against Myocardial I/R Injury by Improvement of Endothelial Function and Activation of AKT1

**DOI:** 10.1371/journal.pone.0038738

**Published:** 2012-08-20

**Authors:** Yoshito Maehata, Shigeru Miyagawa, Yoshiki Sawa

**Affiliations:** Division of Cardiovascular Surgery, Department of Surgery, Osaka University Graduate School of Medicine, Osaka, Japan; Leiden University Medical Center, The Netherlands

## Abstract

**Objectives:**

Activated protein C (APC) has a protective efficacy against ischemia-reperfusion (I/R) injury in several organs. The objective of this study was to investigate effect of APC in myocardium with possible mechanism.

**Methods:**

We used regional and global myocardial I/R injury models of rats. They consisted of I/R injuries (1) by ligation of left coronary artery, or (2) using Langendorff apparatus. Langendorff was used to focus the mechanism of APC excluding coagulation cascade in a working heart. Each experiment had an APC group (n = 10) and a control group with normal saline (n = 10). Injections of these solutions into rats were performed 30 minutes before the planned-I/R injury. Cardiac performance after the procedure was evaluated by echocardiography or indices with Langendorff apparatus. Coronary flow (CF) was measured in the global I/R injury model. Western blotting was performed to detect the change of AKT1 signal in myocardium after global I/R injury.

**Results:**

LV function improved significantly in the APC group: %EF at 2 weeks after procedure, 70.8%±4.5% vs. 56.5%±0.7%; APC vs. control; p<0.01. Percent LV development pressure (LVDP) also improved in the APC group significantly, 88.8%±45.3% vs. 28.1%±15.4%; APC vs. control; p<0.01. In APC group, %CF improved significantly, 88.5%±15.8% vs. 65.0%±13.4%; APC vs. control; p<0.01. It was enhanced significantly when acetylcholine was administered; % CF: 103.5%±9.9% vs. 87.0%±12.1%; APC vs. control; p<0.05. Western blotting revealed that APC significantly induced activation of phosphorylated AKT1 in myocardium (p<0.05).

**Conclusions:**

APC has a novel effect to protect myocardium and cardiac performance against I/R injury through improvement of endothelial function and activation of AKT1.

## Introduction

Standard treatment for ischemic heart disease is revascularization of coronary artery. It is performed by percutaneous intervention or surgical procedure, using cardiopulmonary bypass (CPB). But revascularization induces ischemia-reperfusion (I/R) injury in the regional myocardium. And CPB and cardiac arrest during cardiac surgery can be a cause of global myocardial I/R injury. I/R injury relate to coagulation cascade in abnormal condition, inflammation, endothelial dysfunction, apoptosis and necrosis. To prevent organs from I/R injury, many effective drugs have been discovered by regulating these mechanisms [1], [2].

Activated protein C (APC) is a serine/threonine protease and a physiological anticoagulant. It regulates coagulation system by inactivating coagulation factors Va and VIIIa. And it also possesses anti-inflammatory and anti-apoptotic effect. These factors have been investigated using a variety of organs of animal models with I/R injury [3]–[14]. In particular, some reports focus anti-apoptotic effect of APC against myocardial I/R injury [15]. However details of the mechanism or signal of anti-apoptotic cascade are obscure. Our group has reported that APC improved I/R injury in spinal cord as neuroprotective effect through insulin like growth factor (IGF) and AKT1 cascade. AKT1 is one of signals that prevent from heart failure [16]. Therefore we focused APC and AKT1 signal against myocardial I/R injury in this study. We assessed the effect of APC for heart following regional or global I/R injury model of rat at first. And at next, we investigated the possible mechanisms including AKT1 in the global I/R injury model.

## Materials and Methods

### 1. Animals and drugs

We used male Sprague-Dawley (SD) rats [body weight (BW) 250–300 g] provided by Nihon Dobutsu, Osaka, Japan. All experiments were approved by the animal care committee of Osaka University School of Medicine with ethics. We used recombinant human activated protein C (Anact C®; Teijin Pharmacy, Osaka, Japan).

### 2. Experimental design

We used regional and global myocardial I/R injury model of rat. They consisted of (1) ligation of left coronary artery (LCA) model and (2) isolated heart using Langendorff apparatus model. Langendorff apparatus was used to focus the mechanism of APC. This experiment had no component of coagulation cascade, because the solution in this model was not blood. We considered that LCA ligation model supported clinical situation that was not able to be experimented by Langendorff model alone. Each experiment had 10 rats in APC group and same numbers of rats in control group with normal saline.

### 3. Generation of the regional I/R injury model by LAD ligation

#### Surgical procedure

In APC group, 50 U (approximately 150 U/kg) of APC was injected into the abdominal cavity of each rat through the peritoneum (i.p.) 30 minutes before the procedure for I/R injury. After that, these rats were anesthetized with isoflurane. Endotracheal intubation was performed to them and mechanical ventilation was done during the procedure. After left thoracotomy in the fifth intercostal space, left ventricle was exposed. And transient myocardial ischemia was induced by ligation around LCA. The point of ligation was approximately 2–3 mm away from the origin of LCA, using a piece of 8-0 nylon suture. We confirmed the transient ischemia of LV by finding color of the area turned pale [17]. Ischemic time was 30 minutes. And then blood flow was re-perfused, resulting from removal of suture around LCA. At last, the wound was closed with 4-0 nylon sutures as rapidly as possible. After extubation, we observed rats for 4 hours after surgery. Then we kept these rats for evaluation of cardiac performance for 2 weeks.

#### Evaluation of regional LV function

LV function was evaluated by echocardiography (SONOS 7200®; Hewlett Packard, Loveland, CO, USA). The evaluator was another investigator who had no information about the experiment group, whether APC was used to rats or not. Ejection fraction (EF) (%), fractional shortening (FS) (%), end-diastolic area (EDA) (cm^2^) and end-systolic area (ESA) (cm^2^) were measured in the short axis view at the papillary muscle level, 1 and 2 weeks after the procedure. After that, rats were sacrificed for histological examination. EF and FS were calculated as %EF and %FS, with the values at each time were divided by those before ischemia.

#### Measurement of myocardial infarct area

We made five slices per heart after sacrifice of rat. And we measured infarct area at the level under the papillary muscles. Size of infarction was assessed by hematoxylin-eosin (HE) and Sirius red (SR) staining 2 weeks after the surgery. Staining allowed us to visualize the part of the myocardial infarction with fibrosis. We assessed reperfused area of LCA by injection of blue ink from the ostium of LCA. The size of infarct area was calculated as percentage of reperfused area using Image J (version 1.42).

### 4. Generation of the global I/R injury model by Langendorff apparatus with isolated heart

#### Surgical procedure and determination of LV function

Age-matched male SD rats (BW 250–300 g) were pretreated with heparin (100 U/kg, i.v.), followed injection of pentobarbital sodium. As soon as deep anesthesia had been induced to rats, their hearts were rapidly isolated in ice-slush and mounted in Langendorff system [18]. The method was as follows: the time required for initial stabilization was 20 minutes with perfusion of solution at a speed of 10 ml/min. The solution consists of NaCl, KCl, NaHCO_3_, Glucose, KH_2_PO_4_, MgSO_4_·7H_2_O and CaCl_2_. Several indices those are LV development pressure (LVDP) (mm Hg), the derivative of LV pressure (maximum (+) and minimum (−) dP/dt) (mm Hg/s), heart rate (HR) (bpm) and coronary flow (CF) (ml/min) were measured. +/− dP/dt reflects systolic and diastolic function of LV. After stabilization, the heart was arrested by stop the perfusion. Heart was maintained at 37°C for 30 minutes in an organ bath, filled with a degassed solution. After that, re-perfusion of gassed-solution to the heart was started for 120 minutes. Level of lactic acid dehydrogenase (LDH) in leakage from isolated heart during reperfusion was checked in all collected samples using the SRL© immuno-inhibition method. LDH is an index of myocardial damage.

As in experiment 1, rats in APC group were administered the drug i.p. 30 min before heart excision. The dose of APC was 50 U (approximately 150 U/kg; APC group), whereas controls were administered normal saline i.p.

#### Assessment of endothelial function with %CF

Percent CF was measured as an index of endothelial function. It was calculated as follows: the CF value during reperfusion was divided by that before ischemia [19]. Additional tests with nitroglycerin (NTG) or acetylcholine (Ach) were conducted during reperfusion. We administered each drug to the heart through the aorta 15 minutes after beginning of reperfusion, when their condition was stable. We assessed endothelial function of coronary artery with APC by these CF change.

#### Western blot analysis

A Western blot analysis was performed to investigate changes of AKT1 and phosphorylated AKT1 (p-AKT1) [7]. They are indicators of cell for survival signal [20]. As primary antibodies, goat polyclonal AKT1 (1∶1000; sc-7126; Santa Cruz Biotechnology, Santa Cruz, CA, USA) and mouse monoclonal p-AKT1 (Ser. 473) (1∶1000; 05-669; Upstate, Bedford, MA, USA) were used. Secondary antibodies were horseradish peroxidase-conjugated anti-goat IgG (1∶1000; sc-2020; Santa Cruz Biotechnology) and anti-mouse IgG (1∶1000). The blots were developed using the ECL Plus detection method (RPN2132; Amersham Bioscience Fairfield, CT, USA). A quantitative protein analysis was performed by Image J (1.42 version), followed Western blotting.

### 5. Statistical analysis

All data are shown as mean ± standard deviation. The statistical analysis was performed using an unpaired *t*-test for comparisons between two groups. Differences at p<0.05 were considered statistically significant.

## Results

### 1. Effects of APC in the regional I/R injury model by LCA ligation

All of rats were alive until being sacrificed 2 weeks after the induction of regional I/R injury.

#### Regional LV function

**Figure 1:**
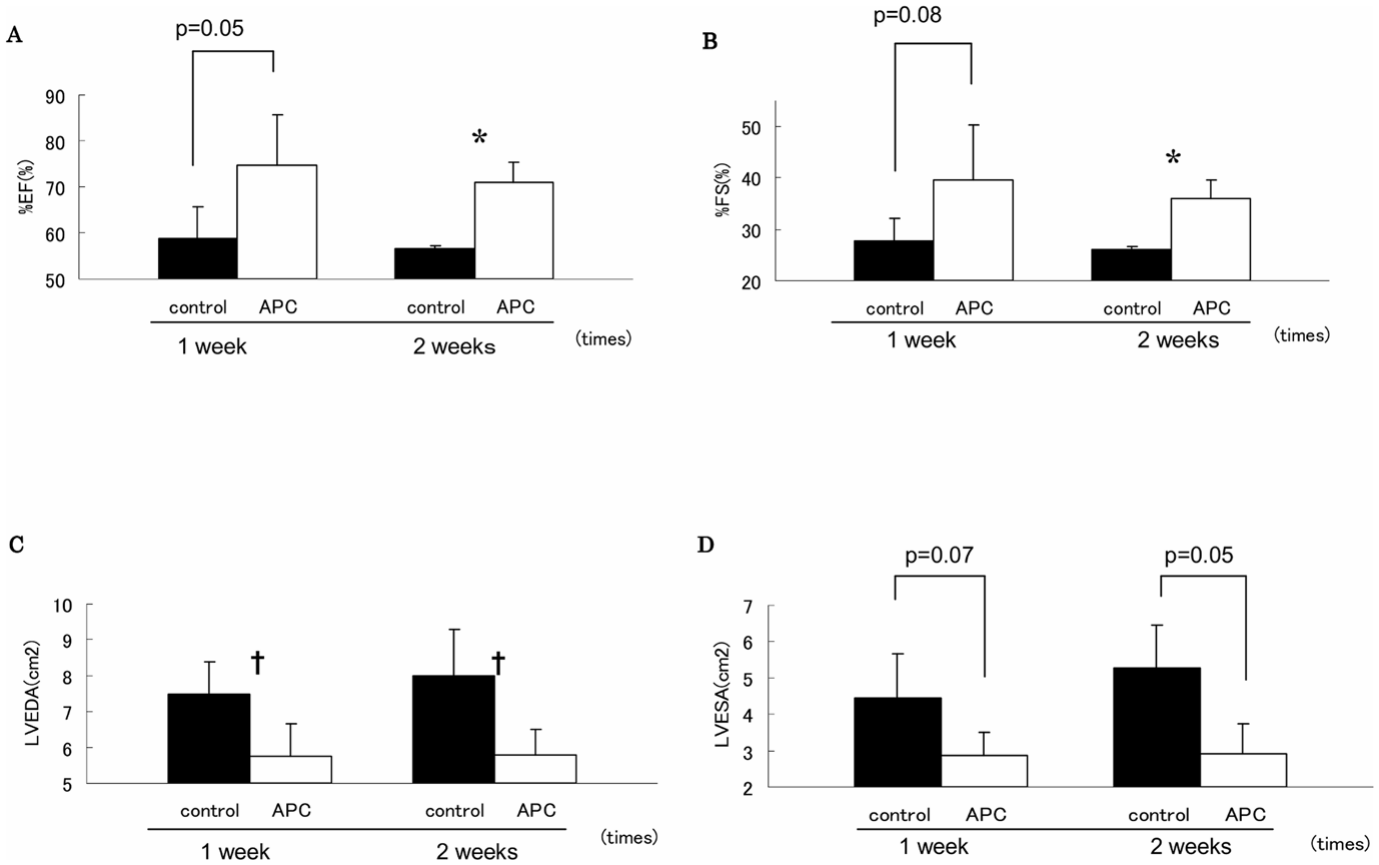
Assessment of cardiac performance in the regional ischemia/reperfusion injury model. We evaluated cardiac function by ultrasound cardiography at 1 and 2 weeks after the procedure in controls and the activated protein C (APC) group (n = 10/group). The parameters were percent ejection fraction (EF), percent fractional shortening (FS), left ventricular end diastolic area (LVEDA) and left ventricular end systolic area (LVESA). Significant differences were observed between the two groups at 2 weeks after the procedure. Open circle indicate APC group and closed circle indicate controls. (*P<0.05; APC group versus controls, **P<0.01; APC group versus controls).

At 1 week after the procedure of I/R injury, %EF and %FS were higher in APC group than controls (%EF: APC group, 74.6%±10.9% vs. controls, 58.8%±6.9%; p = 0.05; %FS: APC group, 39.7%±10.5% vs. controls, 27.6%±4.5%; p = 0.08). LVEDA and LVESA were smaller in APC group than controls (LVEDA: APC group, 5.74±0.92 cm^2^ vs. controls, 7.47±0.93 cm^2^; p<0.05. LVESA: APC group, 2.85±0.65 cm^2^ vs. controls, 4.43±1.25 cm^2^; p = 0.07). At 2 weeks after the procedure, there were more significant differences in cardiac performance between these two groups (%EF: APC group, 70.8%±4.5% vs. controls, 56.5%±0.7%; p<0.01. %FS: APC group 35.9%±3.6% vs. controls, 26.0%±0.49%; p<0.01; LVEDA: APC group, 5.79±0.7 cm^2^ vs. controls, 8.00±1.3 cm^2^; p<0.05; LVESA: APC group, 2.89±0.85 cm^2^ vs. controls, 5.28±1.18 cm^2^; p = 0.05, Figure 1).

#### Size of infarction

**Figure 2:**
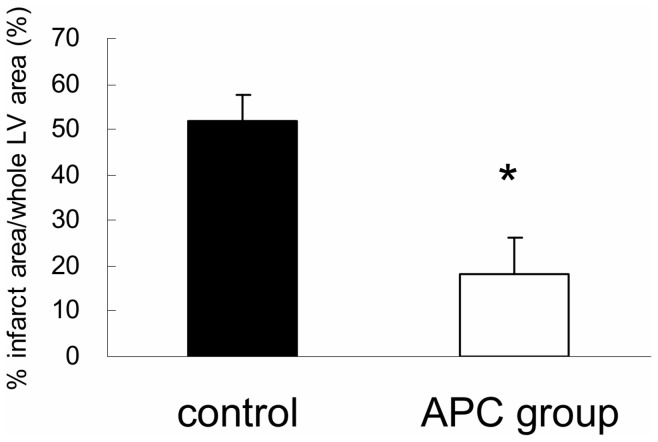
Assessment of myocardial infarct size after regional ischemia/reperfusion injury. We stained slices of left ventricular (LV) tissue with hematoxylin and eosin and Sirius red 2 weeks after inducing transient ischemia. The size of the infarct area was assessed by calculating the percentage of total LV area (% infarction) using Image J. The bar graph shows that the percent infarction in the activated protein C (APC) group was significantly smaller than the controls (* p<0.05; APC versus controls).

Infarction which was induced by LCA ligation were not lost following APC administration. However, size of infarction was significantly smaller in APC group than controls. It was based on HE and SR staining (% infarction area/whole LV area: APC group, 18.3%±7.6% vs. controls, 51.7%±5.8%; p<0.05, Fig. 2).

### 2. Effects of APC in the global I/R injury model by Langendorff apparatus

#### Global LV function

Percent LVDP, % +LV dP/dt, and %−LV dP/dt increased significantly in APC group than controls (% LVDP: APC group, 88.8%±45.3% vs. controls, 28.1%±15.4%; p<0.01. %+dP/dt: APC group, 102.9%±40.4% vs. controls, 23.1%±6.4%; p<0.01. %−dP/dt: APC group, 88.7%±45.8% vs. controls, 20.9%±5.4%; p<0.01) (Table 1). No statistical change was observed between these the two groups for the occurrence of arrhythmia, an index of myocardial damage.

**Table 1:** Assessment of cardiac performance in the global ischemia/reperfusion (I/R) injury model.

Group	Controls	APC group	P value
%LVDP (%)	28.1±15.4	61.5±44.8	<0.01
%+dP/dt (%)	23.1±6.4	39.6±13.2	<0.01
%−dP/dt (%)	20.9±5.4	36.7±12.9	<0.01
LDH (U/L)	9.5±4.2	4.4±2.5	0.08

Percent left ventricular development pressure (LVDP), maximum dP/dt (+dP/dt), and minimum dP/dt (−dP/dt) were used as indices of LV function in the global I/R injury model. Significant differences were observed between controls and the activated protein C (APC) group for all indices (p<0.01). No significant difference was found between the two groups for lactate dehydrogenase (LDH), an index of tissue injury (p = 0.08), but a tendency for a decrease in leakage of LDH from the myocardium after APC administration was observed.

#### Level of LDH during reperfusion

The level of LDH leakage from heart decreased in APC group than controls (APC group, 4.4±2.5 U vs. controls, 9.5±4.2 U; p = 0.08) (Table 1).

#### Endothelial function of coronary arteries

Percent CF increased significantly in APC group than controls (APC group, 88.5%±15.8% vs. controls, 65.0%±13.4%; p<0.01). And %CF after administration of Ach. also increased significantly in APC group than controls (%CF: APC group, 103.5%±9.9% vs. controls, 87.0%±12.1%; p<0.05). In the contrast, there was no difference of %CF after administration of NTG between these two groups (Table 2).

**Table 2:** Assessment of endothelial function in the global injury model.

group	controls	APC group	P value
%CF (%)	65.0±13.4	88.5±15.8	<0.01
%CF with Ach. (%)	87.0±12.1	103.5±9.9	<0.01
%CF with NTG. (%)	109.8±15.0	112.4±8.0	0.74

Percent coronary flow (CF) increased in the activated protein C (APC) group compared with controls (p<0.01). Different vasodilator drugs were administered to investigate the response of enhanced endothelial function. One of them, acetylcholine (Ach), increased %CF only in the APC group (p<0.01, vs. controls), whereas nitroglycerine (NTG) increased %CF in both groups (p = 0.74).

#### Activation of AKT by Western blotting

**Figure 3:**
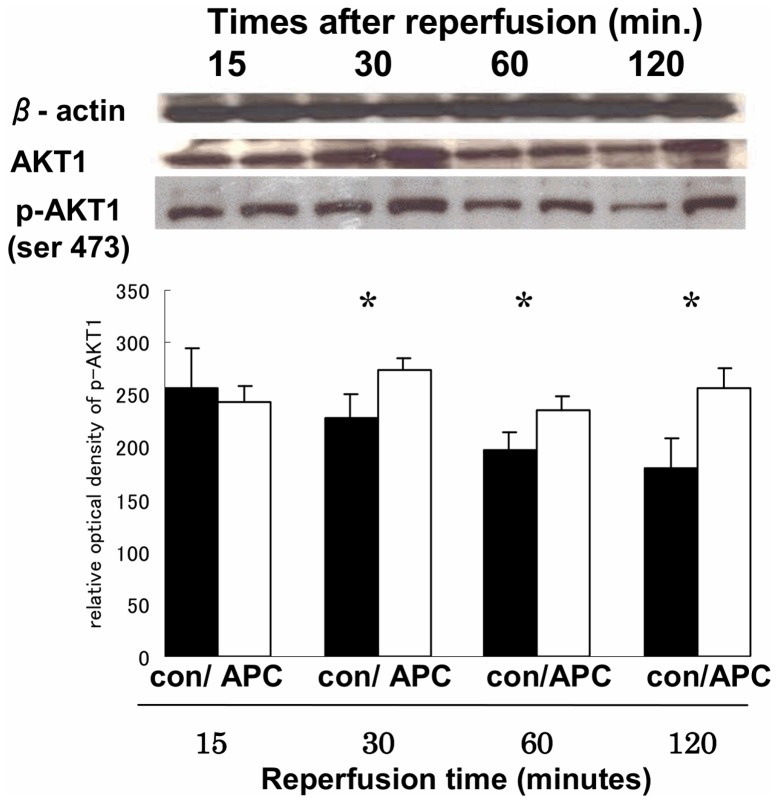
Representative Western blots for AKT1 and p-AKT1. The photograph shows Western blots of myocardium for β-actin, whole AKT1, and p-AKT1in the global ischemia/reperfusion injury model during the time course. AKT1 was strongly detectable 30 min after reperfusion in the activated protein C (APC) group (p = 0.08, vs. control). The bar graph shows that p-AKT1 in the APC group was strongly and significantly expressed 30, 60, and 120 min after reperfusion, (* p<0.05; APC group versus controls).

In APC group, p-AKT1 (phosphorylation at serine 473 residues) was significantly expressed at 30, 60, and 120 minutes after reperfusion than controls (p<0.05 vs. controls) (Fig. 3).

## Discussion

We demonstrated that APC preserved myocardial function against I/R injury in the current study. The experiment with LCA ligation as a regional I/R injury model is nearly to a clinical situation of ischemic heart disease. It revealed that APC improved LV function and decreased infarct area significantly. In the next experiment with Langendorff apparatus as a global I/R injury model without blood component, APC improved endothelial function of coronary artery and induced activation of AKT1 with phosphorylation (Ser 473 residues) significantly. We newly established an efficacy of APC against myocardial I/R injury by improvement of endothelial function and activation of AKT1 in this time.

APC has been useful for treating severe sepsis through its anti-coagulation and anti-inflammatory effects [3]–[14]. It was reported that APC was associated with improvement of cardiovascular function in the Recombinant Human Activated Protein C Worldwide Evaluation in Severe Sepsis (PROWESS) study [21], [22]. Few studies have indicated that APC exerts a protective effect against myocardial I/R injury *in vivo* model with possible mechanism, although I/R injury is major damage that heart can sustain [15].

Some mechanism of APC against myocardial I/R injury *in vitro* model have been reported: anti-apoptosis with reduction of TUNEL-positive cells [21], inhibiting apoptosis and inflammation, which are regulated by protease activated receptor (PAR)-1 [22] and so on. PAR is a receptor of endothelial cell. However, no findings were reportedfor anti-apoptotic signal in detail.

In contrast, a variety of substances or pathways which are triggered by administration of APC have been reported. They are P38 mitogen-activated protein kinase [23], nuclear factor κB [24], tumor necrosis factor-α and p53-mediated pathway [4], [25]. In particular, PI3K-AKT pathway is focused as survival signal during heart failure [26]–[28]. Our group have reported that APC has a neuro-protective effect through AKT cascade after I/R injury of spinal cord in rabbits [9]. Thus we selected AKT1 as one of the possible mechanisms which were induced by APC against myocardial I/R injury. And there was sub-possible cascade with AKT1 in detail. AKT1 is a family of serine/threonine kinases consisting of at least 10 isoforms. It has been reported that different an isoform of AKT1 has counter regulatory on coronary endothelial permeability [29].

In the current study, we used p-AKT1 with serine 473 residues as primary antibody in Western blotting. In addition to this, we performed same experiment using p-AKT1 with threonine 308 residues although we didn't showed in this manuscript. The former experiment showed activation of AKT1 signal with phospholyration after myocardial I/R injury by expression in Western blotting. However the latter one didn't it at all. These results might have a new point of view that APC improve cardiac performance after I/R injury by varied pathways.

In the current study, we also found that APC improved endothelial function as another phenomenon which was related to cardiac performance. Endothelial function is one of the important components maintaining blood fluidity and cardiovascular homeostasis [30], [31]. Of course, activation of AKT1 and improvement of endothelial function induced by APC may be independent factors each others. Finigan et al. reported that APC mediates lung endothelial barrier enhancement with cross talk between endothelial protein C receptor (EPCR) and sphingosine 1-phosphate (S1P_1_) [13]. Feistritzer et al. stated that p-AKT enhances lung endothelial barrier function by affecting cross activation between the S1P receptor-1 and PAR on endothelium [8]. S1P is a potent endothelial cell barrier-enhancing agonist. PAR and EPCR are component of endothelium. They play a critical role in anti-apoptotic cascade which is induced by APC against myocardial I/R injury [5]. According to these previous reports, we might be able to suggest that APC induced a homeostatic effect on coronary flow by improvement of endothelial function with activation of AKT cascade. And we can add the result that p-AKT1 with serine 473 residues is more possible signal in the mechanism of AKT1-endothelial function for cardiac performance, as mentioned above.

### 

#### Study limitations

We were unable to clearly elucidate the overall mechanism that APC protected myocardium against I/R injury [32]. We should further investigate the dose depending on the effect induced by APC. We also did not analyze the activity of a pivotal vasodilator, nitric oxide (NO). Further studies of NO may be needed to develop the mechanism for endothelial function. Especially the correlation between endothelial function and AKT1 should be focused in detail [33].

#### Conclusion

APC has a novel effect to protect myocardium and cardiac performance against I/R injury, possibly through improvement of endothelial function and activation of AKT1.
